# Electricity in the Functional Neuroses

**Published:** 1896-07

**Authors:** A. D. Rockwell

**Affiliations:** New York


### ELECTRICITY IN THE FUNCTIONAL NEUROSES.

BY A. D. ROCKWELL, M. D., OF NEW YORK.

Functional diseases of the nervous system, and indeed all functional derangements, are of far greater importance in the consideration of methods of relief than diseases that are organic or structural. Much has been written about progressive degeneration of nerve tissue, but we still find and, in all probability will ever find, that all our therapeutics avail but little in dealing with gross central or peripheral nerve lesions. A man suffering from a grave organic lesion is very much in the condition of him who is in a state of senility. The house is falling to pieces, the landlord refuses to make further repairs, and unassisted by nature, medical art is practically powerless. With what, however, in our ignorance we term functional diseases, the case is widely different. Nature here does not withhold her aid, and supplemented by the resources of science, the most destroying and persistent functional diseases are successfully dealt with. Of all the forms of the unctional neuroses, neurasthenia is perhaps the most important. Not alone because of its frequency and the suffering it entails, but because the results of the treatment are so much more satisfactory, than in many of the diseases belonging to this class. The following case illustrates both the results of treatment and the difficulty in differentiating sometimes in the selection of the proper form of current.

Mr. E., aged twenty-nine, stated that to the age of twenty-six, he had been to an unusual degree exempt from every form of mental and physical ailment, and his present condition he attributed in part to the influences of heredity, and in part and for the most part, to indiscretions in diet, late hours and sexual excesses. He could still endure physical fatigue, and could at times concentrate his mind, and reason clearly and with forec, but during most of his waking hours, his life was rendered miserable by two symptoms so common among neurasthenics, namely, hopelessness and morbid fears. His mental depression was profound, and by no argument could he be induced to believe there was any hope for him.

A sort of mild panaphobia (fear of everything) existed like a steady undercurrent greatly adding to his distress, although the most prominent feature of his case was a fear of meeting anybody, man or woman, for the first time, and a fear of doing anything that he was told to do or that he thought he ought to do.

This fear so possessed him that it was six months after he had decided to consult me before he came into my presence, although he had come to the door a number of times, going away without ringing the bell, and on one occasion actually entered the waiting-room, taking his leave, however, in great trepidation after sitting a few moments. Symptoms of a less noticeable character were an atonic voice, with occasional disturbances of the functions of the nerves of special sense. I relate this case not only because it illnstrates how well some neurasthenic conditions react to treatment, but also the peculiarity of idiosyncrasy. The faradic current irritated him, whether given locally or generally. Static electricity, which is so grateful in many cases, was even worse in its effects, but from the moment the galvanic current was applied he began to improve. He was remarkably tolerant to the current. Applied by the method of central galvanization with a broad electrode to the head, he bore without discomfort as high as forty milliamperes, while with the electrode at the neck (cilio-spinal centre) seventy-five milliamperes were given.

Under this method of treatment continued for two months the improvement was very great. A moderate degree of cheerfulness took the place of despondency, his morbid fears in great measure disappeared, and he again resumed his work with resolution and hope.

It is this susceptibility or peculiarity of constitution that renders it so difficult in many instances to differentiate between the various manifestations of electricity in any given case. Even after long experience in its use one too frequently finds that the question as to the selection of the proper current can only be determined by a few tentative applications. While this difficulty of differentiation is quite real, and is often the occasion of delay in obtaining results, it must not be supposed that there are not a number of definite indicatious that materially aid in the selection of the proper form of current in the treatment of the functional neuroses.

Take for example the question of pain.

Pain, as the result of an acute inflammatory process, contra-indicates for the most part the use of electricity. This renders it of positive diagnostic value. In cases of severe uterine abdominal pain, the introduction into the vagina of a bi-polar electrode, and the application of a high tension faradic current will increase the pain if it be due to acute congestion, but if the pain be of a hysterical or pseudo-neuralgic character the relief is immediate. In this condition, uni-polar methods of application—by which is meant one pole internally and the other externally—are inefficient. The action of the external electrode on the voluntary muscles induces contractions that are unbearable, while the high resistance of the skin interferes to some extent with its efficient action. Both poles acting, however, through the low resistance of the mucous membrane, and on the comparatively insensitive tissues of the vagina and uterus, makes it possible to utilize a current strength of great power, without the slightest pain or violence of contraction.

The effects of pressure are exceedingly useful in enabling one to select the proper current for the relief of many superficial and even deeply-seated pains. When the pain is increased by pressure, it will be found almost invariably that if electricity is of any service, it will be through the use of the galvanic current.

If on the contrary, firm and equal pressure affords relief, as it so frequently does, the faradic current is, as a rule, far more efficacious than the galvanic. It is in such conditions that the sinusoidal and high tension induction currents are of such value. All this is equivalent to saying that in true neuralgia the galvanic current is indicated, while in false or pseudo neuralgias—which are simply forms of pain, occupying certain areas and running seemingly in the direction of certain nerves—the faradic or the sinusoidal currents are the more useful.

